# Passing the Torch: Reflections on Aging Cell and Translational Geroscience

**DOI:** 10.1111/acel.70569

**Published:** 2026-06-03

**Authors:** Monty Montano

Over the course of my tenure as Editor‐in‐Chief of *Aging Cell*, I sought to help position the journal within a broader transformation occurring across the biology of aging. When I wrote my first editorial, “Aging Cell and the Growing Pains of Translational Geroscience” (Montano 2023), the field had moved beyond a predominantly reductionist focus on isolated molecular mechanisms of aging toward a more translational and integrative framework. Aging Cell is a flagship for fundamental biology, but I aimed to expand the conversation toward how aging mechanisms interact with environmental context, life history, and biological heterogeneity to shape real‐world health trajectories and healthspan outcomes.

Over the past two years the editorials have highlighted that aging is not a singular or uniform process, but rather a highly heterogeneous and context‐dependent phenomenon shaped by genetics, immune function, organ‐specific biology, environmental exposures, psychosocial stressors, and lived experience across the life course (DeGregori et al. 2025; Hinton Jr et al. 2024; Maklakov et al. 2026; Montano 2025; Montano et al. 2024). In this regard, the journal increasingly emphasized the importance of systems‐level thinking and interdisciplinary approaches capable of linking molecular hallmarks to organismal outcomes and population health.

This perspective was reflected in editorials exploring mitochondrial heterogeneity and organelle crosstalk, which argued that the hallmarks of aging cannot be fully understood in isolation from one another, nor apart from the intrinsic and extrinsic factors that shape biological trajectories over time. Rather than viewing aging solely through static molecular pathways, editorials highlighted the importance of biological interconnectedness, environmental context, and adaptive responses across tissues and organ systems (Hinton Jr et al. 2024; Montano et al. 2024).

Similarly, editorials on aging and cancer (DeGregori et al. 2025) emphasized that cancer biology cannot be disentangled from the biology of aging itself. Immune aging, tissue remodeling, frailty, resilience, environmental exposures, and allostatic burden all collectively shape cancer susceptibility, progression, and therapeutic response across the lifespan (DeGregori et al. 2025). Research in this area has reinforced the need for greater integration between geroscience, oncology, immunology, epidemiology, and translational medicine.

Aging Cell has been reframing aging not only as a process of decline, but also as a question of resilience and healthspan. In particular, the emerging framework of immune resilience (Montano 2025) has helped crystallize an alternative perspective, one focused on the biological mechanisms that preserve health, adaptive capacity, and recovery from stress. Rather than asking only what drives disease, we can ask what enables individuals to sustain function and resist deterioration across decades of life. Importantly, Aging Cell has reshaped geroscience around preserving function and extending healthspan across diverse populations and lived environments. This shift toward resilience biology, reserve, and health preservation became an increasingly important intellectual direction for both the field and the journal (Montano 2025; Montano et al. 2024; Ferrucci et al. 2024).

More recently, editorials on ecological perspectives in aging (Maklakov et al. 2026) have further expanded this framework by emphasizing the importance of studying aging within natural and variable environments rather than exclusively within highly controlled laboratory systems. These editorials highlighted how social behavior, ecological pressures, cumulative stress exposures, and environmental variability shape aging trajectories across species and populations, while also underscoring the growing importance of multimodal longitudinal data, wearable technologies, AI‐driven analysis, and real‐world monitoring approaches capable of capturing aging as a dynamic process unfolding across lived environments (Maklakov et al. 2026). In many ways, these approaches also began to democratize geroscience by extending aging beyond highly controlled laboratory and clinical trial settings and toward the diverse biological, social, and environmental realities in which aging actually unfolds.

Taken together, these editorials reflected the evolution of *Aging Cell*. The journal has increasingly expanded from a primary focus on molecular aging mechanisms toward a more translational and integrative vision of geroscience, centered on heterogeneity, resilience, environmental interaction, systems biology, and healthspan across the lifespan. Throughout this period, I hoped to encourage work that bridged disciplinary boundaries and connected fundamental mechanisms of aging to the larger questions of how health is maintained, why aging trajectories differ so profoundly among individuals, and how we might ultimately preserve function and vitality across the human lifespan (Montano 2025; Montano et al. 2024; Ferrucci et al. 2024).

Finally, it has been both a privilege and an honor to serve as Editor‐in‐Chief of *Aging Cell* during this period of rapid growth and transformation in the field of geroscience. I am deeply grateful to the outstanding Deputy Editors, Editorial Board members, reviewers, Wiley publishing team, and colleagues at the Anatomical Society whose dedication, insight, and professionalism helped shape the journal over these past several years. Most importantly, I want to thank the authors and broader scientific community whose rigorous, mechanistically grounded research continues to advance our understanding of aging biology and improve the prospects for healthier aging and extended healthspan. I look forward with optimism and excitement to the journal's next chapter, as well as to the continued evolution of the field itself.

## Author Contributions

The author takes full responsibility for this article.

## Funding

The author has nothing to report.

## Conflicts of Interest

The author declares no conflicts of interest.
